# Why Do Homeless Families Exit and Return the Homeless Shelter? Factors Affecting the Risk of Family Homelessness in Salt Lake County (Utah, United States) As a Case Study

**DOI:** 10.3390/ijerph16224328

**Published:** 2019-11-06

**Authors:** Keuntae Kim, Ivis Garcia

**Affiliations:** Department of City and Metropolitan Planning, University of Utah, 375 South 1530 East, Suite 235, Salt Lake City, UT 84112, USA; ivis.garcia@utah.edu

**Keywords:** family homelessness, homeless shelter exits and returns, subsidized housing programs, Homeless Management Information System (HMIS), survival data analysis, hazard risk, Salt Lake City, Utah, United States

## Abstract

Previous quantitative research on family homelessness has addressed a question of why some households become homeless. However, why some homeless families return the shelter to repeat their homelessness has not been explored well. This study aims at providing a comprehensive insight into the dynamics of homeless families by identifying the physical, social, and economic characteristics of a homeless family affecting the likelihood of their decision to stay, exit, and return the shelter. The relationships of factors with shelter exit and return were examined using Kaplan-Meier estimates of survival times and Cox Proportional Hazard regression analysis. This study employs a sample of 2348 historical records for 1462 homeless families registered to the Homeless Management Information System (HMIS) database between January 1, 2015 and December 31, 2017. The results indicate that structural factors such as subsidized housing program enrollment during a homeless episode and prior income play a significant role in reducing the risks of shelter exit and return rather than physical characteristics of a homeless family. Additionally, results show that variations in prior residence and exit destination of homeless families serve as factors determining the length of their shelter stay and the likelihood to return to the shelter. Integration of both shelter exit and return analysis results make policymakers and urban planners think about developing policies for coordination of housing and economic stability to address family homelessness.

## 1. Introduction

For most people, homelessness is not a permanent condition. Most individuals or families at risk of homelessness overcome their homeless situations within one or two years. Still, some of them remain homeless for an extended period of time because of various reasons—such as disability status, loss of income sources due to the economic crisis, the collapse of their social support networks, trauma from domestic violence, and so on. In the academic literature on homelessness, chronic homelessness has been one of the major issues in urban planning, social work, public health, and sociology. Although many factors are affecting the increase in the risk of homelessness—such as job loss, medical crisis, crime involvement, substance abuse, eviction, and so on—most families can get back on their feet and stay in permanent housing. Nevertheless, annual point-in-time count data by the U.S. Department of Housing and Urban Development indicate that about one-fourth of families that were homeless once are at the high risk of being homeless again [1].

As of 2017, homeless families make up about one-third of the national homeless population in the United States. Over the past decade, change in the national trends of family homelessness has affected how homeless services are planned and delivered. These include an emphasis on addressing the needs of chronically homeless people and a focus on increasing supportive housing, which combines permanently affordable housing with supportive services for persons who need assistance to remain housed. Another national trend in dealing with homelessness includes a “Housing First,” or rapid rehousing approach, rather than a transitional “housing readiness” approach. These approaches incorporate mainstream systems—such as health care, mental health, foster care, and criminal justice—in planning for the prevention of homelessness and meeting the service needs of homeless people. Recent national efforts now include a “coordinated impact approach” that places greater responsibility on government and community-based services working together to address homelessness. As a result of adopting these approaches to address the risk of family homelessness, national figures for homelessness have started to decline over the past decade; For instance, in Utah, the reported point-in-time count for homeless families measured by the Department of Housing and Urban Development (HUD) reached the highest point in 2009, but the number of homeless families began to decline after 2009. The reported point-in-time count for homeless families in Utah dropped by 20.2% between 2015 and 2017, from 1216 to 970.

These national trends of decline in homeless families have been reflected in homeless shelter practices in Salt Lake County (UT, USA). For example, The Road Home (TRH), a major homeless shelter organization in Utah, has adopted various homeless prevention and subsidized housing programs to address homelessness. Like those of homeless shelters across the nation, priorities of the TRH plan include helping homeless people to regain affordable housing quickly, providing appropriate services and housing subsidies for long-term stability, helping people gain employment, and linking homeless families with housing, treatment, and services. In addition to these priorities, several other local concerns and trends contribute to the context for developing a homeless assistance plan that addresses families who return to the shelter after receiving assistance. These include the emphasis on scattered-site housing programs, and anticipated permanent loss of shelter beds, and a nationwide shortage of resources for homeless programs and housing development—notably, the scarcity of resources to support homeless shelters like TRH. Given these national trends of reducing shelter, it is crucial to develop housing search assistance, case management, and other interventions to facilitate the fastest possible transition to permanent housing for families facing repeated homelessness.

In the academic fields—such as sociology, public policy, social work, urban planning, and so on, many studies have been dedicated to understanding why households become homeless in the first place. Nevertheless, there is still a lack of research dedicated to understanding why families return to the shelter. The Housing First initiative and its housing programs have reduced homelessness nationwide. This successful program has missed the mark in reducing the re-occurrence of homelessness on these chronically homeless families. Previous research has explored policy changes and their effectiveness in reducing homelessness. Nevertheless, the TRH, like many other homeless service providers, worries about 30% of families who are returning to the shelter after having received assistance under this new model.

Under these contexts, this study aims at analyzing factors affecting the risks of homeless family’s shelter exit and returns by using microdata on homeless families in Salt Lake City (UT, USA) as a case study. Microdata used in this study includes detailed information on historical records of shelter stays and homeless program enrollment for each homeless family as well as their demographic, social, and economic characteristics. Research questions in this study focus on identifying: (1) which families are exiting or returning to the shelter; (2) which specific strategies—primarily housing programs—can improve hazard risks of shelter exits and returns for homeless families.

The contents of this study are organized as follows: The next section briefly summarizes existing literature on factors affecting family homelessness and revisits methodological approaches used in past studies. Based on methodological approaches identified through the literature review, the data and method sections briefly explain procedures for data processing for the longitudinal data of homeless families in Salt Lake City, Utah, and how to specify and analyze factors affecting the risk of exiting and returning the homeless shelter over time. Interpretation and discussion of analysis results are articulated through a set of tables and hazard risk curves. This study ends with conclusions, limitations, and further research to understand the dynamics of family homelessness.

## 2. Literature Review

### 2.1. Understanding the Dynamics of Family Homelessness

As one of the long-time subjects in urban sociology, interest in exploring the dynamics of homelessness has been gradually grown in parallel to rapid urbanization and development of estimating the homeless population since the 1980s. In the earlier literature, exploring and analyzing the dynamics of homelessness have relied on the changing definition of the term “homelessness” over time. The dictionary’s definition of the term homelessness is people who live on the streets without having their home or places suitable for habitation. However, the operational definition of homelessness in urban sociology research includes a much broader population group—for example, including people who live in the homeless shelter, subsidized housing, hotel and motel vouchers provided by social service providers or stay with their friends’ or relatives’ houses. How the homeless population is defined and counted for analysis influenced policies to decrease the number of the homeless population and allowed for developing targeted homeless service programs [2,3,4].

Due to limited microdata availability, earlier studies focused on describing characteristics of the homeless population and identifying factors based on single-wave survey data at a specific point of time [5,6,7,8,9]. When time-series datasets tracking histories of shelter use and subsidized housing program enrollment for homeless individuals and families became available since the mid-1990s, many studies examined the characteristics of homeless families apart from the homeless population. They found that the dynamics of family homelessness is different from those of single homeless adults [10]. The patterns of homeless families are similar to those of other low-income families or households under poverty. For example, most of the homeless families have a female head who is young, belongs to an ethnic minority group, and has many children. Also, homeless families are suffering from housing instability and have limited mobility [11,12,13].

The fact that housing instability and unaffordability have a significantly negative impact on overcoming family homelessness enabled policymakers to adopt a “Housing First,” or rapid rehousing approach to decrease or end family homelessness. National efforts to address homeless include a “coordinated impact approach” that places greater responsibility on government and community-based services working together to deal with family homelessness. Throughout the existing literature and homeless policies, the dynamics of causal pathways to family homelessness involve complex interactions of individual and structural factors.

### 2.2. Identifying Factors Affecting the Risk of Family Homelessness

Existing literature on homelessness has classified the primary causes of homelessness into two groups—individual and structural factors. Individual factors include demographic characteristics (i.e., gender, age, race, and ethnicity) and personal physical status (physical disabilities, mental health, family dynamics, and abuse experience) associated with the risk of homelessness.

Many earlier studies examined the effects of individual factors on the risk of entering into or returning to homelessness because personal backgrounds or demographic characteristics led to prevent obtaining economic opportunities and maintaining their housing stability. In case studies of New York City and Philadelphia, Culhane and Metraux analyzed the effects of race, ethnicity, age, and gender on the public shelter use. They found that there were significant disparities among age groups and across racial/ethnic groups [14]. Using two-wave longitudinal data, the authors found decreasing trends of the overall shelter use in the two cities. Yet, analysis results also revealed that the public shelter use of homeless families with a female head and children under five years old did not show as a similar decrease as that of single adults. Through multivariate regression models of sample survey data tracking homeless families over time, research suggests that the heterogeneity of demographic characteristics among homeless families is one major component of the pathways to homelessness [15] and determines typologies of homelessness in terms of shelter use—the transitionally, episodically, and chronically homeless [16,17].

A few studies suggest that the effects of physical and mental barriers of a homeless family head on the risk of family homelessness may be another contributing factor to increasing the risk of family homelessness for homeless families belonging to the minority population groups [7,18]. Mainly, mental health problems among homeless mothers or family heads affect risks to mental health and development of children in homeless families [19,20]. Therefore, the effects of promoting mothers’ and children’s mental health on decreasing the occurrence of family homelessness have been examined so far [21,22,23,24].

Literature review studies have summarized the effects of individual factors on the dynamics of family homelessness. These literature review studies argued that the prevalence of family homelessness is higher for young families with small children, and a female household head and African American homeless families tend to remain homeless longer than other race homeless families [13,25,26,27]. Recent studies of the empirical literature on family homelessness argue that the impacts of individual factors on the risk of homelessness were examined in the 1980s and 1990s, but using the longitudinal data of tracking detailed information on homeless episodes for each homeless individual allowed researchers and policymakers to identify another group of factors more directly associated with the dynamics of homelessness (i.e., entering into, exiting from, and returning to homelessness)—that is, structural factors [28,29].

Structural factors are factors associated with the risk of homelessness that measures social and economic conditions of homeless families before entering the shelter and becoming homeless. Many structural factors affecting the risk of family homelessness have been identified in the existing literature. Structural factors include unemployment/employment [4,8,16], income levels [30,31], housing subsidies [32,33,34], education attainment [35,36], and prior residential experience [37,38]. Research suggests that an increase in family homelessness in the U.S. was attributed to increasing rates of income inequality, child poverty, and lack of affordable housing. Weak economic conditions occurred across different spatial levels—national, state, and local levels—made many homeowners in the high-poverty or low-income neighborhoods hard to maintain their housing stability, further abandoning their home to either double up with their relatives/friends or end up with staying at the emergency shelter until they obtain opportunities to move to stable housing with subsidies [8,28].

Longitudinal research since the late 1990s began to include economic condition factors in predicting the probability of homeless families exiting from or returning to homelessness. By investigating structural effects on the risk of homelessness through case studies of large metropolitan cities with data available, many studies now argue that structural factors are more directly associated with the risk and causes of exiting from or recurring family homelessness than individual factors [4,39].

Previous research has attempted to understand the effects of income stability on the risk of family homelessness, but results are quite mixed depending on what dependent variable of interest researchers use to address the dynamics of homelessness. Earlier qualitative research suggested that struggle with stabilizing homeless family’s income level for their self-sufficiency was significantly associated with recovery of homelessness. However, from quantitative research perspectives, some studies found that monthly income level before being homeless was not statistically significant [8,40], while a few studies argue that prior income levels of homeless families played a vital role in predicting the general rates of homelessness [41]. In a typology of family homelessness through case studies of four jurisdictions (Philadelphia, New York City, Columbus, and Massachusetts State), Culhane et al. finds a positive relationship between the mean cost per family and shelter stay—homeless families whose mean cost per family was high prior to entering shelter tend to stay longer in the shelter than the other two groups—temporary and episodic homeless family groups whose mean cost per family is lower than the “long stay” homeless family group [16].

Unlike analysis results on the effects of income on homelessness, much of the literature mentions that lack of affordable housing and housing instability are directly associated with the risk of being and returning to homelessness. At the aggregate level, earlier studies assumed that the rapid increase in homelessness since the 1980s was the result of losing the balance between the number of households below the poverty line and the number of affordable housing units available in the fair housing market [42,43]. Based on this assumption, McChesney suggested the “low-income housing ratio”—a ratio by dividing the number of households below the poverty line by the number of affordable housing units available—and argued that increase in the rate of homeless people could be analyzed in terms of change in the ratio [6].

Many quantitative studies found that homeless families who moved to transitional housing or received housing services with subsidies were less likely to return to the shelter. However, much of the literature also argues that homeless families who received housing services had tendencies to stay longer in the shelter until they got opportunities to enroll in housing service programs [14,39]. Bassuk and Geller reviewed quantitative studies about the role of housing and services on exiting homelessness and confirmed the existing finding that access to housing vouchers or subsidies play a significant role in increasing residential stability [32]. When combined with case management and other services such as job training, homeless prevention programs, and child foster care, housing programs can also contribute to pursuing self-sufficiency and family reunification.

A recent research synthesis study also admits the overall positive impact of housing interventions and housing on exiting family homelessness. Nonetheless, the study argues that this effect may be limited because improving the housing circumstances of homeless families do not necessarily mean that they are residentially stable and economically self-sufficient to support their needs [29]. A few studies attempted to estimate the optimized delivery time of housing interventions and service programs—for example, Metarux and Culhane argue that decrease in the number of repeated family shelter stay days was the greatest when homeless families received subsidized housing less than 180 days (6 months) [9]. However, the modeling results of optimizing the number of days for supporting subsidized housing for homeless families are inconsistent because of the difference in managing these housing intervention programs and lack of information about the nature of the interventions.

Other than income and housing instability, a few recent studies examine the environmental determinism approach to address the pathways of homelessness. For example, after hurricane Katrina, Unity of Greater New Orleans found that increase in the fair-market rent price (from $676 in 2005 to $990 in 2007) forced about 12,000 people to be homeless in New Orleans. A few studies analyzed characteristics of neighborhoods that could produce homeless families and found that neighborhoods with more public housing units, more people who moved in 15 months or less, and more households without telephone service tend to produce the higher number of homeless families than other neighborhoods [44,45].

Qualitative studies have used housing histories to understand the vulnerability of being homeless and identify the reasons people move from place to place [46]. Collecting biographical or in-depth housing life histories has enabled researchers to examine how job loss, medical crisis, incarceration, and other events across the life-span have influenced the likelihood of homeless families being evicted and becoming homeless due to housing instability [47,48,49,50]. Through the careful documentation of housing histories, particular attention is given to address how those who have experienced housing instability or homelessness describe and interpret their experiences [51,52]. Qualitative analysis on housing histories of homeless families not only reveals their difficulties being poor but also explains how being involved in crimes, being a veteran, having a disability, or being a member of a racial or ethnic group might have contributed to their risk of housing instability. These qualitative studies have substantially informed our quantitative analysis—particularly, selecting individual and structural factors to examine the risk of being homelessness and returning to the shelter for another homeless experience.

## 3. Data and Methods

### 3.1. Data

#### 3.1.1. Data Sample

To address the pathway of family homelessness, this study uses the Homeless Management Information System (HMIS) database. As a federal-level and comprehensive homeless database system administered by the U.S. Department of Housing and Urban Development, the HMIS database is the most reliable and valid database that allows us to estimate the risk of homeless families being homeless again. Use of the longitudinal data enables researchers and policymakers to compare the demographic, social, economic status of homeless individuals or families over time and understand the effects of different public interventions—especially subsidized housing program interventions—on reducing the risk of returning to homelessness [53].

Since the HMIS data are highly confidential data that covers detailed historical records of each homeless family registered in the shelter, the authors worked closely with the Road Home shelter to coordinate HMIS data access and manipulation for analysis. Under the permission from the Road Home shelter, the authors were able to obtain the HMIS data for the recent three years—historical records of homeless families registered in the Road Home HMIS database system between January 1, 2015, and December 31, 2017. These two dates also compromise the start and end dates of the periods in this study.

#### 3.1.2. Data Processing for Homeless Family Selection

The initial HMIS data were extracted into eleven relational data tables, and each table was produced at either the client- or program-specific level. These eleven tables need to be joined to one another for further analysis. Fortunately, each table provides several types of identifiers, such as program enrollment identifiers, homeless family identification numbers, and identifiers for assessment of the status of a homeless family. Using these identifiers, the authors wrote R codes that allow querying and joining tables together into one big table by using data table joining techniques such as one-to-one, one-to-many, and many-to-many join.

Based on the homeless family identifiers, 1614 unique homeless families were identified, but there are 141 homeless families having records of entering the shelter before January 1, 2015. Uncertain identification of an initial homeless shelter stay record would cause left-censoring bias—showing less intense shelter use or return patterns in the analysis. Therefore, client- and program-specific HMIS data records for these 141 homeless families were excluded to minimize the effects of left-censoring bias and ensure confidence that homeless families starting an initial spell of their homelessness after January 1, 2015, were used in this study.

Another data processing step before conducting analysis is to define metrics of time in our longitudinal datasets. Suggested by Metraux and Culhane, two types of metrics of time are commonly used for the analysis of shelter use and reentry dynamics in homelessness studies—stay and episode [54]. The term “stay” means a single record of shelter stays. In the HMIS data of this study, one single stay has a unique “program enrollment” identifier. If one or more shelter stays are consecutive, we can group them into one discrete period of shelter use. It is called “a shelter episode.” During a single episode, a homeless family can enroll one or more programs provided by the homeless shelter organization. While aggregating the HMIS records at the homeless episode level, additional eleven homeless family records were removed because of missing information on the date of exiting each homeless stay. After data aggregation at the homeless episode level, the table, including 2348 homeless episode records for the 1462 homeless families is produced for shelter exit analysis.

Similar but separate data processing steps are executed for shelter reentry analysis. To analyze the risk of shelter reentry, previous studies divided homeless family data into one or several cohorts and explored shelter reentry patterns before developing a prediction model for hazard risk of homelessness [35,44]. These studies identified cohorts of homeless families based on their dates of exiting the homeless episode and tracked reentry patterns over the follow-up period. This study adopts this methodology to study patterns of shelter reentry for homeless families in Salt Lake City, Utah. From the 2015–2017 HMIS data, two distinct homeless family cohorts can be extracted such as (1) homeless families exiting the shelter between January 1, 2015 and December 31, 2015 (2015 cohort); (2) homeless families exiting the shelter between January 1, 2016 and December 31, 2016 (2016 cohort). By splitting the HMIS data sample into two cohorts, we were able to investigate reentry patterns over several different periods. For the 2015 cohort, we tracked their reentry pattern over two years; for the 2016 cohorts, we tracked reentry patterns over one year. For both 2015 and 2016 cohorts, we track shelter reentry patterns of both cohorts over a one-year follow-up period. The use of cohort data over different follow-up periods produces three distinct regression models, which allows us to understand the dynamics of homeless family reentry patterns over time, depending on the characteristics of different homeless family cohorts. The data sample size for shelter reentry analysis after data processing includes 1726 homeless episodes for 1140 homeless families, which is about 62.7 percent of the total homeless people throughout Utah State.

### 3.2. Case Study Selection: Salt Lake County, Utah

As the central county of the Salt Lake City Metropolitan Statistical Area (MSA), Salt Lake County is the most populous and urbanized county in Utah, where 1.1 million population live. The population density of the county is 490/km^2^, but the high population and urban density are identified in Salt Lake City which is the capital municipality city in Utah State.

The large population and the non-uniform population density have created a large number of homeless people in the county and Salt Lake City. Utah State has three Continuum of Care (CoC)—a regional or local planning organization that allocates housing and services funding for homeless individuals and families—, and Salt Lake County occupies one independent CoC. According to the 2018 Point-In-Time estimates by the U.S. Department of Housing and Urban Development (HUD), there are 1,804 people experiencing homelessness on a given night, which is about 62.7 percent of the total homeless people in Utah on a given night. Although Utah is one of the U.S. states having the lowest rate of homelessness (9 homeless people per 10,000 people in the general population), the rate of homeless per 10,000 people in the “Salt Lake City & County” CoC is 15.4, which is the largest among the three CoCs in Utah. As with other U.S. States, Utah and Salt Lake County has more female-head homeless families than homeless families with a male head. However, more than two-thirds of homeless families are ones with a White family head, which is different from the other U.S. States. This is because approximately 75 percent of the population in Utah is White. Still, the risk ratio of homelessness is the largest for Black population, which may imply a mixed result of the racial effect on the risk of homelessness.

**Figure 1:**
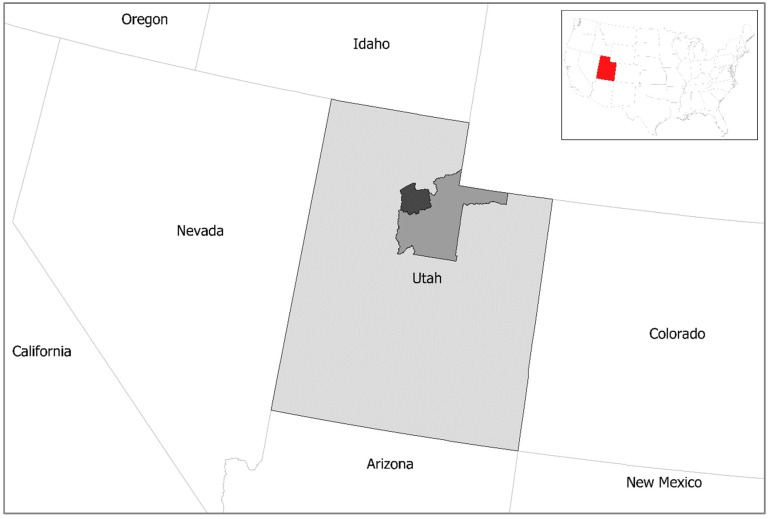
Continuum of Care (CoC) in Utah (Salt Lake City & County CoC in dark gray).

To reduce or end homelessness, the “Housing First” initiative has been adopted across the U.S. since 2007. Utah and Salt Lake County also adopt programs and strategies in the initiative. The “Housing First” initiative suggests four types of housing service programs for homeless individuals and families. They include emergency shelter, transitional housing, rapid rehousing, and permanent supportive housing. Currently, emergency shelter and rapid rehousing are the most common housing options that homeless people frequently use, but the rapid rehousing program has been considered the best option to protect homeless people from being homeless again. Due to the “Housing First” initiative, the total number of homeless people has declined since 2012, but the number of homeless people in families has remained constant over time, suggesting that addressing individual and structural factors affecting the risk of family homelessness allows policymakers to devise policy options or programs for reducing the number of homeless families and ending family homelessness in the long term. Using the HMIS databased of homeless families between 2015 and 2017, this study focuses on identifying the relationships between individual and structural factors and the risk of homeless families exiting their homeless episode and returning to the shelter for a subsequent homeless episode. Figure 1 illustrates three CoCs jurisdictions in Utah. Characteristics of homelessness for three CoCs in Utah are found in Table 1.

### 3.3. Methods

In alignment with previous studies on homelessness, this study uses Cox Proportional Hazard regression and Kaplan-Meier estimates of the survival times to describe and analyze factors affecting the risk of exiting family homelessness and shelter re-entry. Like a difference-of-means test, the Kaplan-Meier estimates of the survival times test difference in the days spent exiting the shelter across different groups, so it will be used to evaluate impacts of various homeless programs or homeless family status on shelter exits or reentry of homeless households. As a specific type of regression model for survival data, a Cox Proportional Hazard model allows us to address questions about the occurrence and timing of events by using longitudinal data whose probability of event occurrence varies over time [55]. A Cox Proportional Hazard model assumes that the covariates (all variables in the model) have the same proportional effects on the ratio as a function of time and event variables. In this study, however, the event variable is about whether a homeless household exits the Road Home program. Unlike an adverse event or status variable used in the model, the event in this study is a positive event—that is, exiting the shelter to overcome or end their homelessness. Therefore, interpretation of the ratio in our model is the proportional effect on exiting the Road Home service program in response to a one-unit change in the corresponding covariates.

While cleaning up the HMIS data, the “time-to-event” and “status” outcome variables were defined to perform survival data analysis using a Cox Proportional Hazard model. Existing studies illustrate an exit from the shelter lasting a certain number of consecutive days, so this study adopts this definition—going out of the Road Home shelter by completing the Road Home programs a homeless household receive and no longer enrolling it. Similarly, re-entry to shelter is defined as returning the Road Home shelter and re-enrolling the Road Home program after exiting the shelter for days. Specifically, this study defines shelter reentry estimates as the risk of returning the shelter after being rehoused through TRH’s housing programs because research questions in this study are about whether and if so when a homeless household returned to the Road Home shelter after being rehoused. Based on these definitions, enroll and exit dates of the Road Home program use were aggregated into episodes of shelter use and allowed calculating the amount of time (i.e., the total number of days throughout episodes) until a homeless household no longer enrolled the program and exited the shelter. The difference in the number of days between episodes is time a homeless family spent outside the shelter until a homeless household returned the shelter. The Cox Proportional Hazard model represents and predicts a change in the likelihood that a homeless household exited the program to overcome their homelessness or returned the shelter after being rehoused through the rapid rehousing (RRH) program, given that a homeless family did not exit the shelter or returned the shelter by the point of time.

## 4. Results

### 4.1. Descriptive Statistics of the HMIS Data Sample

Table 2 shows descriptive statistics for homeless families according to whether they repeated their homeless episodes or not. Among the 1462 homeless families starting their homeless episode between January 1, 2015, and December 31, 2017, the table indicates that approximately 86.5 percent of the data sample was female, and 73.1 percent of homeless families were families with a White family head. 78.6 percent of homeless families were families whose age of a homeless family head was between 20 and 39. The average family size was 3.5, and the number of adults within a homeless family was either one or two adults regardless of repeated homeless episodes. Approximately 48.1 percent of homeless families had a family head with a physical disability.

More than half the homeless families responded to no domestic violence issues before starting their homeless episode, and 7.5 percent of homeless families in the sample data have no information on their local violence experience. Among the 34.4 percent of homeless families reporting their domestic violence, 16.8 percent of homeless families mentioned that they had domestic violence experience more than one year ago. Homeless families with domestic violence issues are more likely to repeat their homeless episodes.

The health status of a family head shows that approximately 24 percent of homeless families had a family head with chronic health problems, while 27 percent had a family head with mental health problems. Overall, family-level sample descriptive results show that homeless families not repeating their homeless episodes seem to be more female, more likely to be White, had a larger family size, and spent more days for their homeless episode than those who repeat their homeless episodes after exits.

Descriptive results from the episode-level sample data show that homeless families were more likely to either double up with their relatives’ or friends’ house or stay at the emergency shelter before their shelter return. The highest percentages of unknown exit destinations are because some homeless families suddenly disappeared while staying at the shelter or other homeless families were still in their homeless episodes. Approximately 20.9 percent of homeless families exited to their rental housing, but 12.6 percent of homeless families exiting their rental housing also returned the shelter for another homeless episode. The average amounts of rental assistance a homeless family received were almost the same regardless of whether a homeless family returned the shelter or not. However, the average total monthly and earned incomes of homeless families returning the shelter tend to be lower than homeless families not repeating their homeless episodes during the study period.

Finally, cohort-based descriptive statistics results show that 48.7 percent of homeless families exiting their homeless episode in 2015 returned the shelter within one year, while 42.4 percent of homeless families exiting the shelter in 2016 returned the shelter within one year. This suggests that the shelter reentry rate slightly declined when tracking historical records of reentry patterns between 2015 and 2016 cohorts over the same follow-up period.

**Figure 2:**
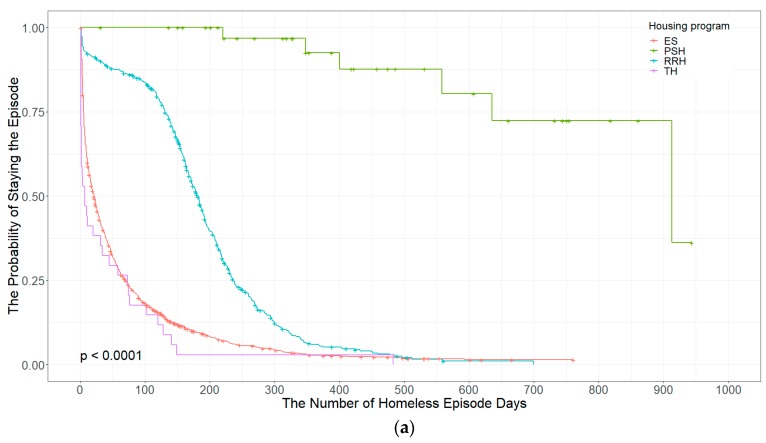

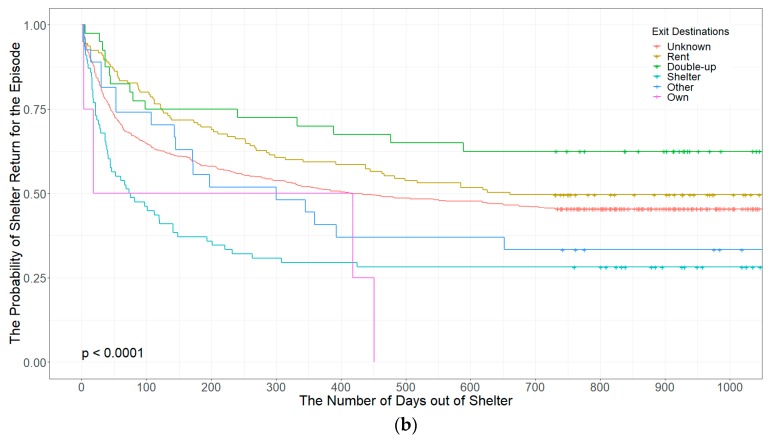
Kaplan-Meier (KM) Curves of the Survival Function: (**a**) Episode Exits; (**b**) Episode Return; Censored data are indicated by the plus sign (‘+’) symbol.

Figure 2 shows Kaplan-Meier (KM) curves for estimates of survival times based on a group. Figure 2a illustrates the probability of exiting a homeless episode depending on the housing program a homeless family spent their time the most during their homeless episode. Homeless families enrolling the permanent supportive housing (PSH) program are more likely to stay in their homeless episodes than any other housing program because it provides housing and intensive services for chronically homeless families with a disability. Among the other three subsidized housing programs, homeless families receiving the rapid rehousing (RRH) program stay their homeless episode longer than homeless families staying at an emergency shelter (ES) or transitional housing (TH). The p-value from the log-rank test also shows that differences in the number of days of staying the homeless episode are statistically significant across all housing programs.

Figure 2b illustrates the likelihood of shelter return for the 2015 homeless family cohort over a two-year follow-up period depending on exit destination types in their previous episode. Except for the KM curve for exiting to housing owned by a homeless family, shapes of the other five KM curves suggest that the probabilities of returning the shelter seem to decline exponentially. These exponential decays of KM curves also show that the hazard risks of shelter return for the five KM curves as gradient functions were high right after the episode exit but became lower as a homeless family spent a longer time out of the shelter. The mean survival time of exiting to housing owned by a homeless family is shortest (222.5 days), while the median survival time of exiting to doubling up with relative’s or friend’s house is longest (630.2 days). The mean survival times of exit destination types for the 2015 homeless family cohort suggests that homeless families exiting to their housing are more likely to return the shelter for their subsequent homeless episode, whereas homeless families exiting to their relative’s or friend’s house are least likely to return the shelter. As with the KM curve for Figure 2a of shelter exits, the p-value less than 0.001 from the log-rank test shows that differences in the number of days out of shelter are significant across the exit destination groups.

### 4.2. Multivariate Analyses

#### 4.2.1. Factors Affecting Shelter Exit

Table 3 presents the Cox Proportional Hazard regression model results for shelter exit. The dependent variable is “shelter exit,” which is a binary variable of whether, and if so, how long a homeless family stayed the Road Home shelter for their homeless episode. Cox proportional hazard regression was applied here to estimate the likelihood of exiting the shelter depending on the number days a homeless family stayed the shelter. Two regression models with different model specifications are implemented, and coefficients of the models represent the hazard ratio, which is defined as the proportional effect of a one-unit change or shift from one category to another in each variable on the likelihood of exiting the shelter. Model 1 shows estimates from a model that includes controls for demographic characteristics and health status of a family head as individual factors of shelter exit. The estimates of each variable in Model 1 suggests that shelter exit is significantly associated with household configuration, health status, and domestic violence. Holding all other variables constant, one additional person in household size or one additional adult is less likely to exit their homeless episode by 9.6 percent and 21 percent, respectively. Having drug abuse and mental health problems also leads to a lower probability of exiting the homeless episode by 26.4 percent for drug abuse and 17.9 percent for mental health. Compared to homeless families having no domestic violence experience, homeless families having memories of domestic violence are less likely to exit their episodes regardless of how long ago domestic violence occurred.

Model 2 shows estimates of a model that includes both individual and structural factor variables. When structural variables are added as controls for the hazard ratio of shelter exit, the household size variable is not significant, but the effects of the number of adults within a homeless family on shelter exit remain significant. Gender and veteran status variables are found to be significant at the 0.05 level; Holding all other variables constant, homeless families with a male family head are less likely to exit the shelter compared to homeless families with a female head. Homeless families whose head of a family is a veteran are 1.5 times more likely to exit the shelter than homeless families without a veteran family head. Having drug abuse problems seems to decrease the likelihood of shelter exit by 28.6 percent. Still, instead of mental health, the estimate of the alcohol abuse status variable indicates that homeless families with alcohol abuse problems are approximately 1.6 times more likely to exit the shelter than homeless families without alcohol abuse issues. Estimates of the domestic violence variable indicate that homeless families experiencing domestic violence at least three months ago are less likely to exit the shelter compared to homeless families without any domestic violence experience. Except for the domestic violence variable, magnitudes of individual factor variables on shelter exit in Model 2 get stronger compared to estimates of the same variables in Model 1.

Among all structural factor variables used in this study, the housing programs a homeless enrolled during their homeless episode play significant roles in decreasing the likelihood of shelter exit. Magnitudes of estimates for each housing program suggest that permanent supportive housing programs decrease the probability of shelter exit among all four housing programs, while transitional housing programs are least likely to decrease the likelihood of shelter exit. Difference in estimates of shelter exit between permanent supportive housing and transitional housing program comes from the characteristics of each housing program; permanent housing programs are targeted to homeless families that need subsidized housing with intensive service care, while transitional housing programs are for homeless families who temporarily are at risk of homelessness or need a short-term stay at subsidized housing.

Prior experience of staying at their housing or doubling up with their relative’s or friend’s houses also affects the probability of shelter exit. Homeless families living in their housing for either one to three months or more than one year are 1.6 and 1.5 times more likely to exit the shelter than homeless families not living in their housing prior to shelter entry. In the case of doubling up, homeless families staying at their relative’s or friend’s houses before entering the shelter are more likely to exit the shelter by 1.2 times to 1.6 times, depending on their length of stay.

Higher levels of specific income sources reported by homeless families affect the likelihood of shelter exit. The HMIS sample data provide detailed information on various income sources reported by homeless families. Among these income sources, having higher levels of average earned income and family support income decrease the probability of shelter exit by about 1.9 and 3.2 percent, respectively. For homeless families enrolling subsidized housing programs, one hundred dollars increase in the average monthly rent assistance decrease the probability of exiting the homeless episode by about 14.5 percent, meaning that homeless families receiving housing with sufficient rental assistance tend to remain enrolled in that housing for their long-term housing stability.

Finally, the categorical variable indicating seasons of entering into their homeless episode is not statistically significant across all seasons. On the other hand, the categorical variable on where a homeless family came from is statistically significant and has a positive effect on increasing the probability of shelter exit. Compared to the reference group of the variable—unknown locations of their origins, comparison of magnitudes of coefficient values between homeless families coming outside and from Salt Lake County suggests that homeless families coming outside Salt Lake County tend to have the higher probability of exiting the homeless episode than homeless families within Salt Lake County.

**Figure 3:**
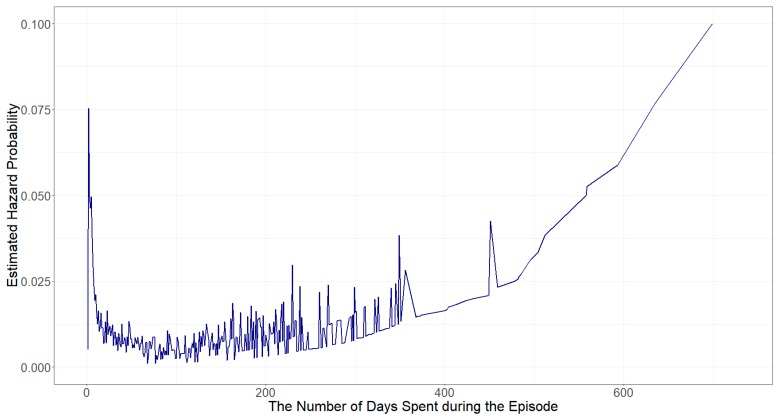
A Hazard Rate Curve for Homeless Episode Exit in Salt Lake City, Utah (2015–2017)

Figure 3 shows a plot of the hazard rate estimated from Model 2—that is, the conditional probability that a homeless family exits their homeless episode, given that a homeless family has not exited their homeless episode yet. Although many noises exist in the graph due to daily hazard rate estimates of episode exit, the “bath-tub” shape of the hazard function curve illustrates that the hazard rates of exiting shelter rapidly decrease in the first 100 days. After the first 100 days, however, the hazard rates gradually increase as the length of the homeless episode increases, meaning that the longer a homeless household enrolls the Road Home program, the higher risk the household exit the shelter by not enrolling the program any longer.

#### 4.2.2. Factors Affecting Shelter Reentry

From univariate survival analysis results from Kaplan-Meier curves of survival function, another set of multivariate Cox Proportional Hazard regression model was implemented to examine the effects of individual and structural factors on the hazard risk of returning the shelter for another homeless episode. Data for shelter reentry analysis are produced by “subsetting” the HMIS sample data based on the memberships of each cohort and follow-up period. Three different survival analysis models are implemented for each homeless family cohort data—the first model for the 2015 cohort over a two-year follow-up period, the second model using the 2016 homeless family cohort over one year, and the third model using 2015 and 2016 cohort data tracking shelter reentry patterns over one year.

Table 4 shows the results of these analyses. In three regression models, the memory of domestic violence a homeless family had before shelter reentry plays a significant role in increasing the hazard risk of returning the shelter for another homeless episode. Compared with the reference category of “No domestic violence occurred,” homeless families experiencing domestic violence are more likely to return the shelter regardless of how long they experienced it. One possible explanation of the highest hazard risk of shelter return for homeless families not reporting their domestic violence may be that there might be many homeless families curtailing or hiding their trauma of domestic violence among that group. In the regression model for the 2016 cohort over a one-year follow-up period, homeless families with a male head are less likely to return the shelter than homeless families with a female head by 46.7 percent, holding all other variables constant. This gender effect on the risk of shelter reentry in the 2016 cohort regression model confirms a consensus that female-headed homeless families tend to be more vulnerable to the hazard risk of becoming homeless again. The 2016 cohort regression model also shows that homeless families having alcohol abuse problems have a lower probability of returning the shelter than homeless families without alcohol abuse problems.

Structural factor variables included in three shelter return models show a significant association with the risk of shelter return. In the regression models for the 2015 cohort over a two-year follow-up period, exit to either housing owned by a homeless family or emergency shelter increases the likelihood of shelter return, while exit to rental housing subsidized by the shelter organization decreases the probability of shelter reentry by 28.5 percent compared to homeless families with no information on their exit destinations. In the all cohort regression model over a one-year follow-up period, homeless families exiting to their relative’s or friend’s houses or subsidized housing are less likely to return the shelter than homeless families with missing information on exit destinations. No significant associations between types of exit destinations and the risk of shelter return are found in the 2016 cohort regression model. However, hazard ratios of average monthly and earned income variables suggest that additional hundred dollar increase in the monthly income increases the likelihood of shelter return by 1.4 percent, while additional hundred dollar increase in the earned income decreases the probability of returning the shelter by 4.4 percent, holding all other variables constant. The similar coefficient trends are identified in income variables of the all cohort regression model, but odds ratios show more significant impacts on the risk of shelter reentry than those in the 2016 cohort regression model.

Finally, in the 2016 and all cohort regression models, homeless families exiting their homeless episode during the winter season between November and February are more likely to return the shelter—by 63.2 for the 2016 cohort and 18.9 percent for the 2015 and 2016 cohort over a one-year follow-up period. The origin of a homeless family in the regression models for the 2015 and all cohorts show a significant association with the risk of shelter reentry; Compared to homeless families coming to the shelter outside Utah State, homeless families whose origins are within Salt Lake County—a jurisdiction of Continuum of Care—are more likely to return the shelter for a subsequent homeless episode by 53.8 percent for the 2015 cohort and 40.5 percent for all cohorts respectively.

**Figure 4:**
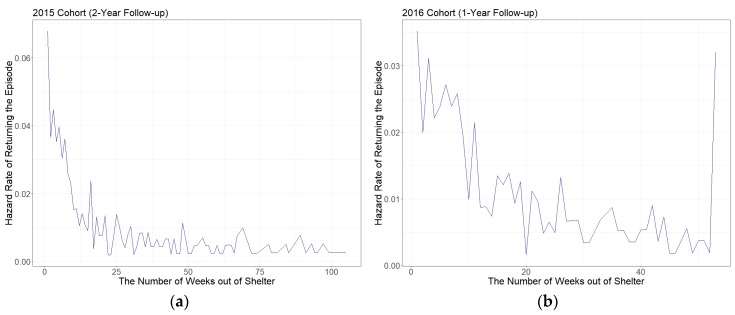

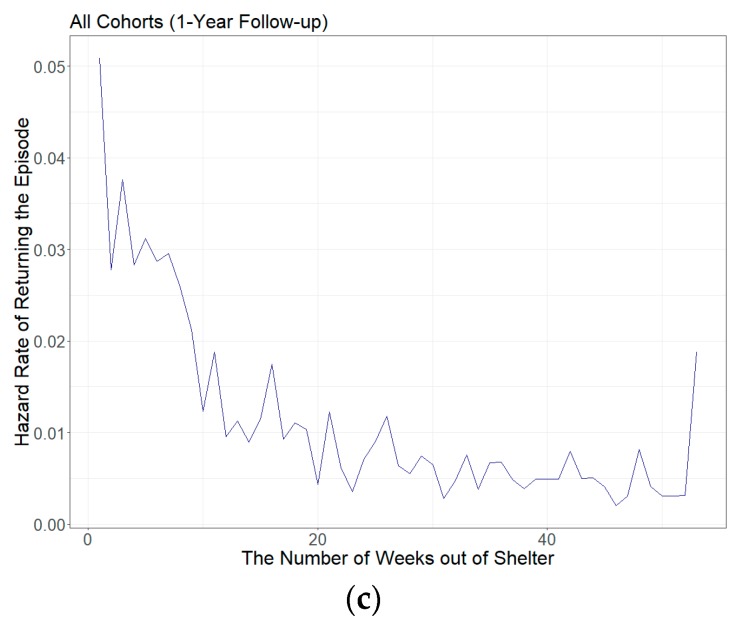
Weekly Hazard Rates of Shelter Return: (**a**) 2015 cohort over a two-year follow-up period; (**b**) 2016 cohort over a one-year follow-up period; (**c**) All cohorts (2015 and 2016 cohorts) over a one-year follow-up period

Figure 4 illustrates three plots of the hazard rates estimated from each shelter return regression model. The hazard rates in these plots represent the conditional probability that a homeless family returns the shelter for their subsequent homeless episode, given that a homeless family is still out of the shelter.

The overall hazard curve trends in three plots show that a relatively high risk of return in the period right after a homeless family exits their previous homeless episode. However, “flat” curves followed by a sharp decline in the hazard risk in the plots suggest that the longer a homeless family stayed out of the episode, the lower risk of returning to the episode they have. A hazard curve of shelter return over two years for the 2015 cohort (Figure 3a) shows a high risk of shelter return right after the exit, a rapid decline in the first 12 weeks (3 months), and a stabilized risk curve after about 20 weeks. A similar hazard curve trend is identified in the all cohort model, but a sharp decline in the hazard rate of shelter return lasts slightly longer (20 weeks) than the hazard rate curve for the 2015 cohort. The overall hazard curve trend for the 2016 cohort (Figure 3b) is similar to the other two curves, but the hazard rates fluctuate much more than the other two curves. Also, the hazard rate of shelter reentry becomes high again three weeks and eleven weeks after the exit. The difference in the Y-axis scale compared to the hazard curves of the 2015 cohort suggests that the overall hazard rates of episode return are relatively lower than those for the 2015 cohort.

## 5. Discussion

### 5.1. Estimates of Time for Staying and Exiting the Shelter

Univariate analysis of Kaplan-Meier (KM) curves for shelter exits indicates that homeless families spending a long time for their homeless episode are more likely to exit their homeless episode. As with existing literature, the decreasing KM curve of estimated survival times—that is, the number of days homeless families staying the shelter—confirms the fact that pressure or motivation for a homeless family to exit the shelter gets higher the longer time they spent in the shelter. However, differences in the median survival times—the number of days staying the shelter for the average homeless family—by types of the housing program clearly show that types of subsidized housing can play significant roles in determining the length of shelter stay of homeless families. Particularly, the KM curves of shelter exit suggest that homeless families under the permanent supportive housing program are the most likely to spend a longer time in the shelter, while homeless families staying emergency shelter for their homeless episode spend the least time in the shelter. For homeless families, a longer stay in their subsidized housing through the housing programs provided by the homeless service provider helps improve their housing stability, which in turn can affect seeking other opportunities to stabilize their life—especially economic stability.

Another KM curves of the shelter reentry rates by different exit destinations in this study supports the fact that subsidized housing program—both permanent supportive housing and rapid rehousing programs—play significant roles in decreasing the rates of shelter returns compared to the other types of exit destinations. Considering that permanent supportive housing is targeted to homeless families who need intensive services due to disability, the KM curve of shelter return clearly shows that a rapid rehousing program is the best option to protect homeless families against the risk of restarting their homeless episode.

Combined with these two findings, this study argues that homeless families receiving subsidized housing tend to spend a longer time exiting the shelter, but homeless families assigned to subsidized housing are less likely to return the shelter for another homeless episode. This is also a clear indication that improving housing stability through the “Housing First” approach works well.

### 5.2. Effects of Individual Factors on Shelter Exit and Return

The multivariate regression modeling results of shelter exits for homeless families clearly show that some individual factors affect the likelihood of shelter exit. Model 1 and 2 of shelter exits in Table 2 indicate that overall homeless household structure rather than demographic characteristics of a homeless family head is directly associated with the probability of exiting the shelter. Although there are disparities in shelter exit between male- and female-head homeless families in Model 2, the multivariate analysis of shelter exits shows that characteristics of family structure such as household size and the number of adults within a homeless family play significant roles in determining their length of shelter stay, which is opposite to findings in earlier studies like Culhane and Metraux (1999) but consistent to those in recent studies. The reason veteran homeless families are more likely to exit the shelter is that the “Housing First” initiative provides a subsidized housing program targeting for veteran families only, and various veteran benefits enable them to exit their homeless situation faster than homeless families without a veteran family head.

Earlier studies in the 1980s and 1990s argued that the relationship between the risk of homelessness and substance abuse was bidirectional [56,57,58,59]. The shelter exit and return models in this study also show mixed results of how substance abuse affects the risk of shelter exit or return, meaning that additional complicated relationships may exist in the causal pathways of the risk of family homelessness. The low probabilities of shelter exit for homeless families having a family head with drug abuse and mental health problems reflect reality that homeless families with drug abuse or mental health problems may have negative impacts on competing for scarce employment and housing opportunities when they are out of shelter—that is, the higher risk of having barriers against maintaining their quality of life after shelter exits. Although there may be several explanations about the relationship between alcohol abuse and the high probability of exiting the shelter, one possible reason would be due to a lack of patience to stay their homeless episode under strict regulations. Also, family heads having alcohol addiction issues frequently cause conflicts with other homeless families or shelter staff, which causes termination of their shelter stays.

### 5.3. Effects of Structural Factors on Shelter Exit and Return

Most of the structural factors included in both shelter exit and reentry models show significant associations with the risk of exiting and returning the shelter for a homeless episode. An independent variable of interest in the shelter exit analysis of this study is how housing affects shelter exit or return for a homeless family, and the shelter exit model indicates that types of subsidized housing program enrollment significantly affect the likelihood of exiting the shelter. Among four different types of subsidized housing programs, a permanent supportive housing program significantly decreases the probability of exiting the shelter, meaning that it would be the best housing option for homeless families to secure their housing stability for a long time and end family homelessness. Given that permanent supportive housing program is targeted to specific homeless families requiring intensive services due to severe disability, coefficients and statistical significance of the other subsidized housing program in the shelter exit model suggests that rapid rehousing program is the best option to provide housing stability to homeless families in general. In the shelter reentry model, impacts of exiting to subsidized rental housing also confirm the fact that subsidized rental housing programs such as rapid rehousing and permanent supportive housing play significant roles in reducing the risk of returning to the shelter for another homeless episode.

Some types of prior residence a homeless family stayed before entering the shelter affect the probability of exiting the shelter. As previous studies found, staying at an emergency shelter or hotel or motel with vouchers does not significantly affect the risk of exiting the shelter. In the shelter exit model, statistically significant prior residence types are housing owned or rented by a homeless family and doubling up with their relative’s or friend’s house. Having experience of staying privately-owned or privately-rented housing for a short period of time or staying in their housing for a long time gives a homeless family strong motivation to overcome their homeless situations and go back to their normal life, which leads to increase the likelihood of exiting the shelter. In the case of the doubling-up, having relatives or friends who can provide housing motivates homeless families into ending their homelessness, which suggests the importance of encouraging community-based actions and social support networks to end family homelessness.

Another critical structural factor affecting the likelihood of exiting and returning the shelter is prior to income status. However, the interpretation of results regarding its influence on the shelter exit and return models is quite mixed. Although eligibility for receiving a housing subsidy is determined regardless of prior income status, having experience of earning money through employment can give homeless families motivation to be less dependent on the shelter stay. An increase in family support income and rental housing assistance can provide homeless families with economic stability to end their family homelessness. However, given that the average monthly income of a homeless family is mostly based on subsidized income resources such as family support income and rent housing subsidies, significance of the average monthly income in the shelter reentry model suggests that excessive dependence on subsidized income sources may lead to repeating their homeless episodes, not helping them overcome their homelessness.

## 6. Conclusions

There have been many studies on family homelessness to identify factors affecting their decisions and behaviors during a homeless episode, but the research that analyzes the relationships between their physical and socioeconomic characteristics and the risk of both shelter exit and return is not widely studied due to limited microdata availability. Using historical records of homeless families registered to the HMIS database during the recent three years, this study contributes to taking both individual and structural factors affecting family homelessness into account for understanding the dynamics of patterns of shelter exits and returns for homeless families. Shelter exit and cohort-based shelter reentry analyses in this study confirm three things: First, structural factors like change in socioeconomic status rather than individual physical characteristics are primary interventions to determine the length of shelter stay and the time a homeless family spends before returning the shelter. Second, this study confirms the finding of previous literature that subsidized housing programs play significant roles in addressing family homelessness effectively. Third, adding structural factor variables to the regression model changed statistical significance levels of gender, veteran status, substance abuse, and experience of domestic violence, which implies variations in the effects of structural factor variables depending on some individual factor variables. Overall, this study confirms the empirical fact that the “Housing First” approach and strategies work well for family homelessness in the U.S.

In addition to confirmation of findings in previous literature on family homelessness, this study examined the effects of new structural factors on the risk of shelter stay and return—that is, income status and the length and experience of prior residence. Due to detailed information on income sources and prior residence of a homeless family over time, this study argues that programs for supporting the economic stability of a homeless family are also necessary to end their homeless situations. Particularly, shelter reentry modeling result of income status in this study suggests that providing homeless families with opportunities for making earned income rather than depending on subsidized income sources is a key to help homeless families maintain their quality of life after exiting the shelter. Integration of results of homeless episode exits and shelter reentry is another contribution of this study to understand the dynamics of family homelessness from quantitative perspectives. Although this study only uses the HMIS data from one region—Salt Lake County in Utah, findings about the impacts of different subsidized housing programs on the likelihood of shelter exit and return for homeless families might be helpful to understand the effects of housing service programs in other states because these housing service programs have been implemented based on guidelines of the “Housing First” initiative. However, differences in statistical significance and magnitudes of some individual and structural factors in regression models of this study suggest that geographical generalizability of the likelihood of shelter exit and return can be achieved through additional HMIS data collection from other states in the U.S.

Despite these findings, this study is subject to several limitations. First, while processing the HMIS data, there are incomplete historical records of some homeless families found in the database. These records were deleted from the regression analysis. Second, this paper does not include some factors such as educational attainment and crime records as factors affecting family homelessness. The Road Home HMIS database does not contain these data, so this study could not examine the effects of educational attainment and criminal status on the risk of being homeless. Controlling for additional variables may make shelter exit and reentry analyses more solid than the models in this study, but education attainment and criminal records are somewhat challenging to catch accurately because homeless families tend to curtail or hide education and crime records. Third, due to limited data availability, historical records of homeless families in Salt Lake County, Utah were used as a single case study for measuring the risk of family homelessness, so additional family homelessness data from the HMIS in other states would help develop generalizable findings of the dynamics of family homeless across the U.S. as a whole. Finally, this paper does not account for variables that monitor the life of homeless families between their homeless episodes. Measures on activities and behaviors of homeless families between shelter exit and subsequent shelter reentry may provide more insights into why they return the shelter. There are a few qualitative studies on the life of homeless families after exiting the shelter, but quantitative approach to analyze this potential association requires the development of a monitoring system of homeless families exiting the shelter, which would be a future research topic on homelessness.

## Figures and Tables

**Table 1:** Characteristics of Homelessness of Three CoCs in Utah (2018).

CoC Name	Total	Homeless Characteristics		
		Sheltered	Unsheltered	Homeless Households
Salt Lake City & County CoC	1804	1688	136	177
Utah Balance of State CoC	899	660	239	91
Provo/Mountainland CoC	173	128	45	19
**Total**	2876	2476	400	227

Source: U.S. HUD Point-In-Time Estimates (2018).

**Table 2:** Descriptive Statistics Results.

Individual and Structural Variables	Episode Repeaters	Episode Non-Repeaters
*n* (1462)	512	950
Characteristics of a homeless family head		
Gender (%)		
Female	31.1	55.4
Male	3.9	9.6
Race (%)		
White	24.8	48.3
Black	5.0	7.5
Indian/Native American	3.6	5.0
Asian/Pacific Islander	1.6	4.2
Hispanic (%)	10.3	17.0
Age when entering the shelter (%)		
Under 20	0.5	0.8
20–29	12.5	22.2
30–39	15.7	28.2
40–49	4.8	11.0
50–59	1.4	2.7
Over 60	0.1	0.1
Disabling condition (%)	18.3	29.8
Veteran (%)	0.8	1.7
Health status (%)		
Alcohol abuse	0.9	1.7
Chronic health	8.8	15.2
Drug abuse	1.7	4.5
Mental health	9.6	17.4
Homeless family characteristics		
Family size (%)		
1	0.9	1.3
2	7.7	14.6
3	11.6	22.6
4	7.5	13.1
5	4.5	6.7
More than 6	2.9	6.6
The number of adults (%)		
1	17.2	32.8
2	17.0	29.8
3	0.8	2.0
More than 4	0.1	0.3
Children under five years old (%)		
0	13.0	25.5
1	14.8	26.3
2	4.9	10.3
3	1.8	2.4
More than 4	0.5	0.5
Domestic violence occurred (%) ^1^		
Unknown	7.3	0.2
No violence occurred	15.1	43.0
Less than 3 months ago	4.2	7.1
3–6 month ago	1.1	1.9
6 month-1 year ago	1.3	2.0
More than 1 year ago	6.0	10.8
The average number of days per episode (days)	84.1	98.3
**Episode-level descriptive statistics (*n* = 2348)**		
The number of episodes	1398	950
Prior Residence		
Unknown	6.6	3.1
Housing by a homeless family	3.9	3.2
Double-up	25.0	16.7
Shelter, hotel/motel vouchers	15.7	10.4
Others	8.3	7.1
Exit destination		
Unknown/Still in the episode	33.9	22.9
Rental housing by a homeless family	12.6	8.3
Housing owned by a homeless family	0.7	0.1
Double-up	3.8	4.1
Emergency shelter	7.1	3.9
Others	1.5	1.1
Average rent assistance during the episode (dollars) ^2^	949.62	946.21
Average total income (dollars)	572.28	591.23
Average earned income (dollars)	153.76	227.42
Reentry rates of 2015 and 2016 cohorts		
2015 cohort (*n* = 833)		
Within 1 year (%)	48.7	-
Within 2 years (%)	55.2	-
2016 cohort (*n* = 893)		
Within 1 year (%)	42.4	-

^1^ The reference group for the domestic violence variable is an “unknown” category. ^2^ This is an average amount of rental assistance for homeless families who received rental assistance through subsidized housing programs from the homeless shelter.

**Table 3:** Cox Proportional Hazard Regression Model Results for Shelter Exit^1^.

Variables	Model 1	Model 2
**Individual factor variables**		
Male	1.064 (0.073)	0.775 * (0.075)
Race		
White	1.075 (0.103)	0.853 (0.105)
Black	0.949 (0.115)	0.843 (0.118)
Indian/Native American	1.004 (0.120)	0.881 (0.124)
Hispanic ^2^	0.969 (0.051)	0.970 (0.052)
Age in shelter entry	0.998 (0.003)	1.002 (0.003)
Household size	0.904 *** (0.020)	0.973 (0.021)
Adults	0.790 *** (0.047)	0.772 *** (0.050)
Children less than 5 years old	1.051 (0.031)	1.044 (0.032)
Veteran ^2^	1.217 (0.157)	1.504 * (0.159)
Disability ^2^	1.039 (0.054)	0.993 (0.055)
Alcohol abuse ^2^	1.343 (0.144)	1.637 ** (0.148)
Chronic health ^2^	0.977 (0.058)	0.912 (0.059)
Drug abuse ^2^	0.736 ** (0.095)	0.714 * (0.100)
Mental health ^2^	0.821 *** (0.057)	0.912 (0.058)
Domestic violence occurred ^3^		
Unknown	1.079 (0.065)	1.100 (0.067)
Less than 3 months ago	0.884 (0.074)	0.975 (0.075)
3–6 month ago	0.655 ** (0.134)	0.789 * (0.069)
6 month–1 year ago	0.675 *** (0.122)	0.711 ** (0.136)
More than 1 year ago	0.661 *** (0.068)	0.683 *** (0.069)
**Structural factor variables**		
Housing program enrollment during the homeless episode		
Emergency shelter		0.463 *** (0.098)
Rapid rehousing		0.210 *** (0.060)
Permanent supportive housing		0.016 *** (0.370)
Transitional housing		0.520 *** (0.181)
Prior residence		
Emergency shelter/hotel, motel vouchers ^4^		
Less than 1 month		1.087 (0.074)
1 month–90 days		0.999 (0.105)
90 days–1 year		0.942 (0.147)
More than 1 year		1.546 (0.260)
Housing by a homeless family ^4^		
Less than 1 month		1.611 (0.508)
1 month–90 days		1.618 *** (0.232)
90 days–1 year		1.115 (0.135)
More than 1 year		1.573 ** (0.140)
Double up ^4^		
Less than 1 month		1.264 ** (0.066)
1 month–90 days		1.044 (0.096)
90 days–1 year		1.128 (0.125)
More than 1 year		1.663 ** (0.132)
Average monthly income ($100)		1.008 (0.007)
Average earned income ($100)		0.981 * (0.008)
Average family support income ($100)		0.968 ** (0.010)
Average rental assistance ($100)		0.855 *** (0.011)
Season when entering the episode ^5^		
Summer (June to August)		1.041 (0.062)
Fall (September to November)		1.045 (0.066)
Winter (December to February)		1.024 (0.066)
Coming from Salt Lake County ^6^		
No		1.356 *** (0.074)
Yes		1.192 *** (0.057)
*n*	2348	2348
R^2^	0.082	0.531
Log Likelihood	−14,409.76	−13,622.00

* *p* < 0.05; ** *p* < 0.01; *** *p* < 0.001; ^1^ The dependent variable here is “shelter exit”—a binary variable representing whether a homeless family stayed the shelter or not. ^2^ The reference group is a “no.” ^3^ The reference group is “No domestic violence occurred.” ^4^ The reference group is “not staying before entering the shelter.” ^5^ “Spring” is a reference group for the season of the shelter entry variable. ^6^ “Unknown” is a reference group for the variable.

**Table 4:** Cox Proportional Hazard Regression Model Results for Re-entry to Shelter ^1^.

Variables	2015 Cohort (2-year Follow-Up)	2016 Cohort (1-year Follow-Up)	All Cohorts (1-year Follow-Up)
**Individual factor variables**			
Male	1.032 (0.159)	0.533 * (0.223)	0.842 (0.132)
Race ^2^			
White	1.003 (0.232)	0.853 (0.105)	0.930 (0.174)
Black	1.409 (0.268)	0.843 (0.118)	1.166 (0.200)
Indian/Native American	1.114 (0.271)	0.881 (0.124)	1.105 (0.204)
Hispanic ^3^	1.133 (0.113)	1.135 (0.115)	1.155 (0.082)
Age when exiting the shelter	0.999 (0.007)	1.010 (0.008)	1.002 (0.005)
Household Size	1.060 (0.045)	0.987 (0.046)	1.016 (0.032)
Adults	1.196 (0.109)	1.036 (0.113)	1.119 * (0.078)
Children under 5 years old	1.042 (0.063)	1.075 (0.073)	1.058 (0.049)
Veteran ^3^	0.632 (0.467)	1.514 (0.434)	0.858 (0.329)
Disability ^3^	0.972 (0.119)	1.284 (0.132)	1.148 (0.088)
Alcohol abuse ^3^	0.635 (0.391)	1.103 (0.418)	0.775 (0.292)
Chronic health ^3^	0.980 (0.127)	0.926 (0.139)	0.961 (0.095)
Drug abuse ^3^	1.136 (0.226)	0.469 * (0.288)	0.829 (0.179)
Mental health ^3^	1.013 (0.127)	0.756 (0.140)	0.878 (0.095)
Domestic violence occurred ^4^			
Unknown	3.176 *** (0.126)	3.035 *** (0.135)	3.125 *** (0.093)
Less than 3 months ago	1.504 * (0.170)	1.386 (0.189)	1.473 ** (0.130)
3–6 month ago	1.758 * (0.272)	2.015 * (0.278)	1.749 ** (0.201)
6 month–1 year ago	1.804 * (0.260)	1.794 * (0.265)	1.787 ** (0.189)
More than 1 year ago	1.579 ** (0.161)	1.672 ** (0.170)	1.612 *** (0.121)
**Structural factor variables**			
Exit Destinations ^5^			
Owned housing	3.379 * (0.530)	1.284 (0.530)	1.074 (0.422)
Rent housing	0.715 * (0.142)	0.821 (0.131)	0.711 *** (0.094)
Double-up	0.757 (0.270)	0.903 (0.168)	0.758 * (0.138)
Emergency shelter	1.435 * (0.160)	1.080 (0.184)	1.240 (0.118)
Other	1.091 (0.255)	0.470 (0.393)	0.742 (0.218)
Average monthly income ($100)	1.019 (0.011)	1.014 ** (0.007)	1.018 *** (0.005)
Average earned income ($100)	0.975 (0.015)	0.956 ** (0.016)	0.966 ** (0.011)
Winter season exit ^3^	0.914 (0.114)	1.632 *** (0.116)	1.189 * (0.081)
Locations ^6^			
Unknown	1.619 * (0.210)	1.389 (0.211)	1.449 * (0.152)
In Utah, but outside Salt	1.573 (0.266)	0.570 (0.382)	1.127 (0.217)
Lake County			
Within Salt Lake County	1.538 * (0.193)	1.272 (0.193)	1.405 * (0.142)
*n*	833	893	1726
R^2^	0.163	0.164	0.138
Log Likelihood	−2859.51	−2400.55	−5509.08

* *p* < 0.05; ** *p* < 0.01; *** *p* < 0.001; ^1^ The dependent variable is “re-entry to shelter,” which is defined as the number of days between the exit date of the previous homeless episode and the start date of the subsequent homeless episode; ^2^ Asian/Pacific Islander is the reference group; ^3^ The reference group is “no.”; ^4^ “No domestic violence occurred” is the reference group; ^5^ The reference group is “Unknown.”; ^6^ “Coming outside Utah” is the reference group.
